# Understanding Safety in Online Mental Health Forums: Realist Evaluation

**DOI:** 10.2196/75320

**Published:** 2025-06-27

**Authors:** Paul Marshall, Neil Caton, Zoe Glossop, Steven Jones, Rachel Meacock, Paul Rayson, Heather Robinson, Fiona Lobban

**Affiliations:** 1 Spectrum Centre for Mental Health Research, Division of Health Research, Lancaster University, D01, Health Innovation One, Sir John Fisher Drive, Lancaster, LA1 4AT, United Kingdom, 44 01524522187; 2 Division of Population Health, Health Services Research & Primary Care, University of Manchester, Manchester, United Kingdom; 3 School of Computing and Communications, Lancaster University, Lancaster, United Kingdom

**Keywords:** digital mental health, peer-to-peer support, social networking, discussion forum, web-based discussion

## Abstract

**Background:**

Online forums are used widely to facilitate mental health peer support. However, concerns exist regarding potential harm associated with their use, and little is known about forum safety from the user’s perspective.

**Objective:**

This study sought to understand how users experience safety within online mental health peer support forums. Following previous research, safety was conceptualized with reference to both experiences of harm and feelings of interpersonal safety within the forum environment.

**Methods:**

Data were collected from 42 semistructured realist interviews and 504 cross-sectional survey responses from users of 3 UK-based online mental health forums. These included a forum hosted by a health service provider with subforums for anxiety, depression, and eating disorders; a corporate provider focused on young people’s mental health; and a voluntary sector provider with subforums for general mental health support, eating disorders, and postpartum psychosis. A bespoke survey was used to obtain descriptive quantitative data regarding user perceptions of forum safety. Qualitative data were used to refine an initial program theories framework comprising context-mechanism-outcome configurations related to forum safety developed in previously published realist synthesis.

**Results:**

Survey responses revealed that over half of the participants felt safe to post because of online anonymity (n=202, 40.1% agreed and n=97, 19.2% strongly agreed), while a minority reported encountering distressing forum posts (n=95, 18.8% agreed and n=18, 3.6% strongly agreed) and expressed concern that talking about mental health online could make them feel worse (n=113, 22.4% agreed and n=17, 3.4% strongly agreed). Refined program theories highlight: (1) the disclosure-promoting effect of anonymity, related to the mitigation of concerns that users’ mental health experiences could be linked to their offline identities; (2) the importance of proactive content moderation for addressing emerging safety issues; (3) a need for organizations to implement rule enforcement sensitively and balance between conversational openness and restricting topics likely to cause distress; (4) forum users’ experiences of self-moderating their exposure to potentially distressing online content; and (5) how the perceived nonjudgement, authenticity, and similarity of other forum users generates interpersonal safety.

**Conclusions:**

This is the first realist evaluation to directly assess processes underpinning safety in online mental health forums. A key novel finding of this study is that safety emerges not only from harm reduction procedures but also from a facilitative interpersonal atmosphere defined by sensitive moderation and the sharing of lived experiences. Hosts should therefore remain attentive to both potential risks and opportunities to foster connections between community members.

## Introduction

Online forums represent an increasingly popular format for mental health peer support [1,2]. Typically organized around particular mental health challenges or demographic groups, online forums facilitate text-based, asynchronous discussions through which users exchange information and support [3]. Third-sector organizations and mental health providers use online forums to extend service reach, and a substantial body of peer-led forums leverage social media to host supportive communities [4]. The advantages of online mental health forums include their accessibility, with forums usually available when needed and free of charge, and the opportunity for users to access forums anonymously, which may promote the disclosure of personal difficulties [5]. There is some evidence indicating that using online forums may lead to positive mental health outcomes [6,7]. However, concerns exist regarding the safety of delivering mental health peer support within an increasingly complex online environment [5]. In this study, forum safety refers to two aspects of user experience identified in prior research [5]. The first relates to the experience of harm, encompassing emotional harm from exposure to distressing content and physical harm if users act on information such as self-harm instructions or health misinformation. The second refers to interpersonal safety—also referred to as psychological safety [8]—reflecting users’ comfort with sharing mental health experiences within the community.

Three salient issues pertaining to forum safety are evident in existing literature. First, a central tension in the delivery of online forums relates to evidence that in some contexts, reading about and discussing distressing experiences can be beneficial, yet in other circumstances, access to conversations about mental health may cause harm. Research with forum users shows how relatable lived experiences can normalize and validate previously stigmatized experiences [9,10], yet research also highlights the possibility that reading descriptions of mental health challenges can be distressing, discouraging, or overwhelming [11,12]. A key consideration for the implementation of online mental health forums therefore lies in promoting constructive engagement while simultaneously reducing the potential for user distress. To this end, forums typically implement rules restricting discussions of particularly emotionally activating content, such as crisis situations or self-harming behaviors, and use moderators to help users navigate this balance [13]. Organizations may also develop protocols for helping users experiencing significant distress, by, for example, signposting to other services or offering one-to-one support [14].

Second, organizations seeking to deliver safe mental health forums must contend with the unique challenges imposed by the online context. For example, the asynchronous, anonymous nature of forum communication can confound attempts to rapidly identify and address safeguarding issues, such as users experiencing suicidal distress, particularly where forums do not require participants to share their personal details at sign-up [15]. Furthermore, user attempts to post rule-breaking or harmful content are widespread in online communication and can include offensive posts or health misinformation [16]. As with forums in domains other than health, organizations may also be required to plan for “toxic” online behavior that could undermine forum safety, such as spamming, predatory users, and attempts by banned users to create multiple accounts to disrupt forum functioning [17]. Tools such as automatic word blockers support some teams with content moderation [18], however, much of this work is conducted manually and requires significant resources [19].

Third, forums aim not only to limit access to harmful content or malicious actors but also to establish a supportive community that feels sufficiently safe to allow users to read and post about mental health experiences. A thorough understanding of forum safety must therefore account for both harm reduction strategies and factors likely to facilitate willing engagement and disclosure. This aligns with literature that conceptualizes psychological safety as a group climate in which people feel able to take interpersonal risks in pursuit of personal goals [8]. There is some evidence to suggest that the intention to post in online forums is associated with perceived psychological safety and that this feeling of safety is underpinned by high trust in community members and low self-consciousness regarding posting [20]. Indeed, research with service users points to the importance of forum characteristics such as a welcoming and caring interpersonal atmosphere in facilitating engagement with online mental health forums [21,22].

Despite the large body of literature on the positive and negative effects of online mental health forums, research focused specifically on user perceptions of safety is limited. This gap is significant given the growing number of online mental health peer support forums and users internationally, and broader social and policy interest in online harms as indicated, for example, by efforts in the European Union and the United Kingdom to develop legal frameworks that promote safety in online user experience [23,24]. To address this understudied topic, we aimed to understand user safety in online mental health forums using a mixed methods approach based on the principles of realist evaluation.

## Methods

### Study Setting and Design

This study is one element of a larger investigation of UK mental health communities called the Improving Peer Online Forums (iPOF) project. As per the iPOF research protocol [25], this realist evaluation represents the second stage of the project, which builds on a realist synthesis conducted in stage 1 [5]. Realist evaluation involves iteratively developing and refining program theories, or causal statements regarding how interventions work, as data collection progresses [26]. Following realist methodology, program theories from the realist synthesis pertaining to forum safety were used to inform this study. These program theories took the form of context-mechanism-outcome (CMO) configurations. CMOs are a primary unit of analytic output in realist studies and seek to elucidate how a mechanism, defined as a response to resources offered by an intervention, occurs within a particular context [27]. Contexts represent factors impacting the activation, or strength of activation, of a mechanism and exist at a range of levels including individual, organizational, social, and economic factors. Outcomes typically refer to cognitive, emotional, or behavioral consequences that follow from the activation of a mechanism. A total of 8 of the 22 CMOs on mental health forum impacts developed in the prior realist synthesis related to forum safety [5]. CMOs from the previous review described a range of forum features and experiences pertaining to user safety, such as the importance of forum moderation, the value of anonymity, and negative experiences emerging from forum use [25]. These CMOs were used to inform an initial program theory (IPT) framework (Table 1) for this study and influenced data collection, as described below.

**Table 1.** IPTs^a^ related to forum safety from the iPOF^b^ realist synthesis [5].

IPT #	CMO^c^ configuration
IPT 1	Posts detailing personal experiences of potentially harmful behaviors (eg, self-injury and restrictive eating; context) that frame them as helpful (mechanism—resource) may normalize and reinforce their use (mechanism—response), increasing the likelihood of users adopting these behaviors (outcome).
IPT 2	When seeking support for issues that others may find distressing (context), users are more likely to post in forums that have ways to flag the potentially distressing nature of their experiences (eg, tags, trigger warnings, or a separate subforum; mechanism—resource). This provides reassurance that posts will not inadvertently cause harm to others (mechanism— response), increasing the likelihood that users will use the forum to seek support (outcome). Other users are less likely to be exposed to distressing content (mechanism—response), reducing potential distress in the wider community (outcome).
IPT 3	For users making an original post (context), the absence of a response or responses that are unrelated to the original post (mechanism—resource) will prompt feelings of being ignored or misunderstood (mechanism—response). This leads to increased isolation (outcome) and reduces forum engagement (outcome).
IPT 4	Those yet to post to forums may be concerned about feeling exposed or receiving negative responses if they share their experiences (context). Observers who see others receiving constructive and respectful responses (mechanism—resource) will be reassured of the safety of posting to the forum (mechanism—response), increasing the likelihood that they will actively participate in discussions (outcome).
IPT 6	Negative social consequences of discussing mental health difficulties, including shame and stigma (context), are overcome by forum anonymity (mechanism—resource), which disinhibits (mechanism— response) users from discussing their experiences, leading to greater self-disclosure (outcome).
IPT 7	Because users’ personal identities are hidden (context), they are insulated from the negative social consequences of rule-breaking (mechanism—resource). This can have a disinhibiting effect on some users (mechanism—response), making them more likely to engage in antisocial behavior such as bullying (outcome), and reducing safety for other users (outcome).
IPT 8	Open online forums with no ways to flag distressing content, poor moderation, or lenient rules (context) are more likely to expose users to posts detailing users’ highly distressing circumstances, misinformation, and “toxic” discussions (mechanism—resource), which can contribute to distress (mechanism— response) and disengagement from the forum (outcome).

a IPT: initial program theory.

b iPOF: Improving Peer Online Forums.

c CMO: context-mechanism-outcome.

Participants were recruited from 3 UK-based mental health forums partnered with the iPOF project. These forums were chosen from the 7 forums in the iPOF study as they represent 3 different service contexts, namely the UK National Health Service, corporate, and voluntary sectors. While only 3 forums were included in this phase of the research, the remaining 4 forums also represent these same sectors. It is hoped that this cross-sector sampling promotes the transferability of the findings of this study to a broad range of service settings. All forums had a mental health focus, could be accessed using anonymous usernames, and had dedicated moderation teams. Key forum characteristics are described in Table 2 and detailed descriptions of each forum partnered with the iPOF project can be found elsewhere [28]. As per an ethical framework developed by the iPOF team [29], forums are described using randomly allocated bird names to protect the anonymity of participating organizations and forum users. Participants were initially invited to participate in the iPOF project via an online survey advertised on forum home pages and through emails from forum hosts to forum users. Those who completed the survey were given the option to consent to be invited to participate in an individual interview.

**Table 2.** Characteristics of included mental health forums.

Forum name	Forum setting	Availability and access	Area of mental health focus	Moderation
Magpie	A large, health-based social networking website with subforums focused on specific health challenges.	Publicly available, 24/7. Posting requires an account.	Four subforums used for recruitment to this study focused on general mental health support in the United Kingdom, maternal mental health support, support for postpartum psychosis, and support for eating disorders.	Forum moderation is conducted by trained volunteers and typically operates within daytime hours.
Dunnock	A large, dedicated platform for mental health support aimed at young people between the ages of 10 and 25 years (note that only those aged 16 and older were eligible for inclusion in this study).	Available 24/7 but requires an account to view and post.	General mental health and well-being.	All user-generated content is reviewed by a trained and paid moderation team before going live on the forum.
Sparrow	A dedicated platform for mental health support hosted by a regional service provider in the UK NHS^a^.	Available 24/7 but is only available to mental health service users under the care of the host NHS service.	Subforums focus on support for depression, anxiety, and eating disorders.	Forum moderation is conducted by a team comprising health professionals, paid and trained moderators, and trained volunteers, and typically operates within daytime hours.

a NHS: National Health Service.

### Quantitative Data Collection and Analysis

The survey used in this study was an online questionnaire designed specifically for the iPOF project. It included 95 items across sections investigating forum use, perceptions of forum helpfulness, moderation, user mental health, use of other health services, and forum safety. Most items, including those reported here, were developed for the purpose of this study by the research team in collaboration with a public and patient involvement group to assess concepts and experiences identified by the iPOF realist synthesis. The questionnaire also includes validated measures of anxiety and depression. A full description of the survey is available elsewhere [30]. Only items directly related to forum users’ perceptions of safety or distress are reported here. This included (1) “I have felt distressed by some of the posts I have read on this forum,” (2) “I feel safe to post about my personal experiences in this forum,” (3) “I feel safe to say anything in this forum because no-one knows who I am,” and (4) ‘Talking about my mental health experiences in the forum might make me feel worse.” In this study, cross-sectional survey data were used to assess the prevalence of user-reported distress and feelings of safety, providing context for the program theories presented thereafter.

### Qualitative Data Collection and Analysis

Participants who had completed the iPOF survey and expressed interest in a qualitative interview were invited to participate. Interviews were conducted via phone or Teams (Microsoft Corp) by PM and ZG, audio recorded, transcribed, and anonymized. The interview procedure followed the principles of realist interviewing [31]. Here, interviewers seek to examine existing program theories by introducing key concepts and ideas that participants are invited to reflect on based on their own experience of the program. Interviews therefore include a combination of deductive questions based on program theories and more exploratory, inductive questions, leaving open the possibility that new concepts not previously captured by the IPT framework may emerge. In addition to the IPTs, interviewers used a semistructured topic guide based on the concepts from these program theories (Multimedia Appendix 1).

Data extraction and analysis occurred simultaneously and followed three stages. First, data segments were extracted into a bespoke data extraction form based on the IPT framework. The form was hosted on Excel (Microsoft Corp) online to facilitate collaborative working. Each IPT was written out in full in a separate column. Data segments were copied under the most relevant program theory along with an analytic comment stating how the data segment contributed to the refinement of the respective IPT. Data extraction was conducted by ZG and PM. Second, data segments and analytic comments from across participant interviews were reviewed against the IPT framework. Particular attention was paid to where data supported, refuted, or refined IPTs, and new CMO configurations were formulated to account for the accumulating evidence. For example, IPT 8 refers to how a lack of moderation can contribute to negative outcomes. In interviews, however, participants reflected on the value of proactive moderation, leading to the development of a CMO configuration reflecting this aspect of their experience. IPTs for which there was insufficient evidence in the interview data to support meaningful iteration, such as IPT 1 regarding exposure to self-harm content, were not included in the subsequent analysis. Initial reformulation of the CMOs was conducted by PM. Following this, interim CMOs were reviewed in a full-day analysis meeting held by PM, ZG, FL, and HR, during which the CMO configurations were further refined with respect to illustrative data segments. Third, the analysis was shared with and reviewed by the wider iPOF team comprising academics, clinicians, and experts with experience in mental health. A final draft of this study was shared with an expert by experience whose perspective on this research is captured in a lived experience commentary presented at the end of this study. Lived experience commentaries are short pieces, typically written independently of the research team, that provide the opportunity for critical commentary from those with lived experience of the research topic [32].

### Ethical Considerations

This study received ethical approval from the Solihull Research Ethics Committee on June 20, 2022 (IRAS314029). All participants provided written informed consent. Interview data have been anonymized. Participants were compensated with £30 (US $40.31) shopping vouchers for completing interviews and £10 (US $13.44) shopping vouchers for completing surveys.

## Results

### Overview

Semistructured qualitative interviews were conducted with 42 forum users from Magpie (n=16), Dunnock (n=10), and Sparrow (n=16) between August 2023 and October 2024. A total of 504 survey responses were collected from Magpie (n=110), Dunnock (n=287), and Sparrow (n=107) between July 2023 and March 2024. Participant demographics are available in Tables3  4. Quantitative data are reported by survey item (Table 5). A total of 5 CMOs were refined and are reported with reference to illustrative interview data.

**Table 3.** Survey participant demographics.

Characteristics	Value, n (%)	
Sex		
Female	344 (68.3)	
Male	115 (22.8)	
Nonbinary	29 (5.8)	
Preferred not to say	11 (2.2)	
Self-defined	5 (1)	
Age group (years)		
16‐24	227 (45)	
25‐34	83 (16.5)	
35‐44	51 (10.1)	
45‐54	41 (8.1)	
55‐64	61 (12.1)	
65 and older	39 (7.7)	
Preferred not to say	2 (0.4)	
Ethnicity		
White	430 (85.3)	
Black	17 (3.4)	
Asian	21 (4.2)	
Mixed	22 (4.4)	
Prefer not to say	8 (1.6)	
Self-defined	6 (1.2)	

**Table 4.** Interview participant demographics.

Participant code	Age group (years)	Sex	Ethnicity
D^a^002	16‐25	Female	Preferred not to say
D003	16‐25	Female	White/White British
D043	16‐25	Female	White/White British
D046	16‐25	Female	White/White British
D073	16‐25	Preferred not to say	White/White British
D090	36‐45	Female	White/White British
D196	26‐35	Female	White/White British
D229	16‐25	Male	White/White British
D458	16‐25	Transgender male	White/White British
D646	36‐45	Female	White/White British
M^b^003	66‐75	Female	White/White British
M007	56‐65	Male	White/White British
M008	46‐55	Female	White/White British
M022	76‐85	Female	White/White British
M032	26‐35	Female	White/White British
M073	56‐65	Female	White/White British
M077	56‐65	Female	White/White British
M100	55‐65	Female	White/White British
M126	46‐55	Female	White/White British
M178	26‐35	Female	White/White British
M213	26‐35	Female	White/White British
M214	66‐75	Female	White/White British
M304	56‐65	Female	White/White British
M321	46‐55	Female	Mixed/Multiple ethnic groups
M326	56‐65	Female	White/White British
M342	56‐65	Female	White/White British
S064	36‐45	Female	White/White British
S^c^119	56‐65	Male	White/White British
S052	26‐35	Female	White/White British
S072	26‐35	Female	Other ethnic group
S002	56‐65	Female	White/White British
S003	66‐75	Female	White/White British
S006	46‐55	Female	White/White British
S011	36‐45	Female	White/White British
S012	36‐45	Female	White/White British
S023	36‐45	Male	White/White British
S031	76‐85	Male	White/White British
S083	66‐75	Male	White/White British
S084	56‐65	Male	White/White British
S019	46‐55	Female	White/White British
S025	26‐35	Female	White/White British
S035	26‐35	Female	White/White British

a D: Dunnock.

b M: Magpie.

c S: Sparrow.

**Table 5.** Survey responses to safety-related items.

	Item 1: “I have felt distressed by some of the posts I have read on this forum,” n (%)	Item 2: “I feel safe to post about my personal experiences in this forum,” n (%)	Item 3: “I feel safe to say anything in this forum because no one knows who I am,” n (%)	Item 4: “Talking about my mental health experiences in the forum might make me feel worse,” n (%)
Strongly disagree	65 (12.9)	17 (3.4)	16 (3.2)	59 (11.7)
Disagree	185 (36.7)	52 (10.3)	53 (10.5)	171 (33.9)
Neither agree nor disagree	141 (28)	151 (30)	136 (27)	144 (28.6)
Agree	95 (18.8)	214 (42.5)	202 (40.1)	113 (22.4)
Strongly agree	18 (3.6)	70 (13.9)	97 (19.2)	17 (3.4)

### Survey Responses

Survey responses revealed that over one-fifth of participants felt distressed by posts they had read (n=95, 18.8% agreed and n=18, 3.6% strongly agreed), indicating that even in moderated communities, a notable minority face challenges with exposure to forum content. Similarly, a quarter of respondents agreed (n=113, 22.4%) or strongly agreed (n=17, 3.4%) that discussing their mental health online could make them feel worse. Feelings of safety when posting was comparable to perceptions of safety due to online anonymity, with 42.5% (n=214) agreeing and 13.9% (n=70) strongly agreeing they felt safe to post, and 40.1% (n=202) agreeing and 19.2% (n=97) strongly agreeing they felt safe to share because of anonymity. This underscores the central role of online anonymity in fostering forum engagement.

### CMO1: Anonymity and Disclosure

#### CMO Configuration

##### Context

Stigma related to mental health difficulties, in particular, concerns that disclosing mental health difficulties within in-person settings or to people users know offline may expose them to negative judgment by others.

##### Mechanism

The ability to post to a forum without revealing personal information (resource) mitigates concerns that judgment by others could be linked to posters’ offline identities (response) and provides users with a greater degree of choice (response) regarding the extent of the information they share online.

##### Outcome

These responses promote the candid disclosure of personal difficulties for those who wish to post.

### Illustrative Interview Data

Anonymity disinhibited users from engaging in online conversations. Several users noted that by not having an online profile linked to their name, they were able to avoid perceived stigma, and therefore, judgment related to their mental health.

*...the stigma around mental health is still hugely prevalent, so to actually be there as someone who’s anonymous that you can actually say how you’re feeling when you write something down.*

[S002]

*I prefer being anonymous myself because there might be people on there that maybe I work with, I don’t know, or a family member. I don’t want them to know the details of why I’m on there.*

[S011]

By offering users the opportunity to engage anonymously, they are given control over what information they share about themselves, and therefore, how they are perceived online.

*Interviewer: ...what makes Magpie feel like a safe place to discuss really personal issues*?

*Interviewee: Because I’m not using my own name ... so nobody knows it’s me, nobody knows where I live unless I choose to tell them so yes it’s that sort of ... you’re anonymous but you’re not*.

[M100]

Similarly, the following user notes that a forum that does not require their profile to include identifying information provides freedom to decide what to disclose.

*...I felt that you have the freedom to give your own information if you want to, but you don’t have to. I relate because I give my age, I give my gender, I give basic things because I think then I get better answers*.

[M304]

### CMO2: Proactive Moderation

#### CMO Configuration

##### Context

The nature of online communication involves interacting with or reading posts by unknown others who could, depending on the nature of forum moderation, share anything, including content that could be disturbing, disagreeable, or inaccurate.

##### Mechanism

Proactive moderation, where moderators are seen to work quickly and sensitively to address potential and emerging safety issues (resource), both limits exposure to distressing content and provides reassurance that the space is supervised (response).

##### Outcome

This promotes perceptions of community safety and users’ willingness to engage with the forum.

### Illustrative Interview Data

Interviewees identified the presence of moderators as an important resource for maintaining a positive community culture and ensuring that distressing content does not discourage users from engaging with the forum.

*...for the safety of everybody someone will review it [a forum post] before it gets published ... to make sure there’s nothing triggering ... if someone put something triggering on there then people won’t want to use them [online forums].*

[D046]

Issues moderators managed included interpersonal difficulties that could arise in forum conversations. As the following participant identified, their presence also provided reassurance.

*...just knowing that it’s moderated, and some of the comments that come through it’s all a very supportive space. You don’t see anybody dismissed or you know threatened.*

[M213]

Participants also highlighted the importance of moderators’ attentiveness to situations where users feel unsafe due to their mental health. One user valued the ability to request support from the moderation team where this difficulty emerged.

*...another really important thing is it’s a hundred percent safe, it is moderated and it is ensured that everyone is safe ... any time someone’s in danger or someone might be just like concerned about someone, if they’ve said they’re unsafe or they feel anxious then they get a message from the moderator just to make sure that they’re okay so it’s really inclusive and overall it is really safe.*

[D2]

Highlighting the importance of proactivity, situations where moderators missed distressing content were identified as unhelpful.

*I have noticed some comments that are like maybe the moderators haven’t noticed them or not really read them properly. It kind of feels like it’s a bit unhelpful in a way and it’s not really a supportive comment. When that happens, we have this option to message the team, and we just let them know and the team removes the comments.*

[D2]

### CMO3: Balanced Rules

#### CMO Configuration

Note that this CMO configuration includes 2 different responses and outcomes to the same resource, representing what in realist methodology is referred to as rival theories [33].

##### Context

In pursuit of safe and supportive online environments, online forum guidelines typically restrict some forms and topics of communication. However, some restricted topics may be personally significant to individual users. Forums must therefore achieve a balance between openness and the enforcement of boundaries within their communities.

##### Mechanism

When implementing forum guidelines, moderators may take action to restrict conversations by editing posts, removing conversations, or restricting access by certain forum users (resource 1). When personally important experiences have been censored, users may feel undermined, particularly where communication by forum staff is seen as unnecessary or harsh (response 1). However, where community rules and moderators’ decisions are communicated sensitively (resource 2), users are more likely to accept moderators’ actions (response 2).

##### Outcome

Feeling undermined through moderator censorship reduces the feeling that the forum is a safe space to discuss mental health experiences and motivation to engage with the forum (outcome 1). However, accepting moderators’ actions leads to users continuing to engage with the forum and abide by community guidelines (outcome 2).

### Illustrative Interview Data

Participants reflected on how restrictions on discussions around certain topics can be valuable for creating a supportive environment.

*People can talk about their daily activity but there’s rules where you can’t talk about how much you eat or weigh ... that’s quite helpful for people who have anorexia or bulimia and it’s good because everyone supports each other*.

[D046]

However, when implemented in a manner that feels rigid and insensitive, forum users could experience significant distress.

*I put in the post that basically I felt particularly terrified because I was sure I’d just eaten about 250 calories ... I got an email back which really upset me at the time basically from one of the staff moderators ... to quote her words,* “*As an opportunity to demonize calories because this is not helpful to other service users,*” *I felt like I was at school, and I was being told off ... I was in a vulnerable state, in a state of considerable distress and it made it far worse*.

[S019]

One user identified that this could have a muting effect on users’ willingness to engage openly with the forum.

*...you need to be careful with what you’re saying and you can tell that sometimes in some people’s stories that they’re posting of how careful they’re being and that doesn’t make me feel very listened to or heard because ... and I don’t know how that other person is feeling but I kind of feel like we’re, the group, we’re being not listened to fully*.

[D229]

Reflecting on the challenge of balancing restrictions with open dialogue, the following participant recognized the need for an approach that charts a path between forum openness and moderator control in the service of forum safety.

*...the (forum manager) thing where only his wisdom is allowed to be discussed, so you don’t want to go too far in either direction. You’re really got to be in the middle where you’re allowed to have a free flow of information and people can talk to each other openly without feeling intimidated by others or intimidated by the moderators. There are two versions of making people feel unsafe. It can go both ways you know*.

[M214]

Some forum users recognized rule enforcement to be an essential element of forum safety. Where moderators do intervene, doing so in a way that communicates the intention to support the user may mitigate possible negative reactions.

*They [moderators] would jump in and say to them, “We don’t post that particular sort of thing here. We hope you’re okay and here’s how we can help.” It’s like shutting it down but shutting it down in a friendly way. Like not telling the person off or making them feel like they’ve done something really bad because quite often we can do things when we’re in distress that we wouldn’t do if we weren’t in crisis ... it’s good if the moderators can kind of say, “I’m really sorry,” or “everyone’s probably really worried about you,” but also in a really caring way because sometimes if you say, “We don’t post that here and we just delete it,” then that comes across as really cold and might actually make the situation worse for them*.

[D229]

### CMO4: Self-Moderation

#### CMO Configuration

##### Context

Online forum posts are user-generated and refresh continually such that forum users do not know the exact nature of the content they are likely to come across. Users’ preferences for the type of content they wish to read or avoid are likely to fluctuate over time with their own mental health. This creates a risk that users may be distressed by something they read online.

##### Mechanism

Design features that notify users of the nature of the content they are about to access, such as topic tags, warnings, or descriptive thread titles (resources) to enhance users’ ability to choose whether and when to access forum content (response).

##### Outcome

This reduces the likelihood that users access forum posts that distress them.

### Illustrative Interview Data

Some interviewees noted that their experience of safety in an online forum could be undermined by unexpected and particularly emotionally charged content.

*...crazy ideas and very explosive comments ... if you start reading some of the comments ... so I’m talking about the safety from the point of view of your illness ... okay, suddenly I’m being disturbed and stirred up. Your emotions, if you read something which instead of helping you it can actually make you feel worse, so that’s what I’m saying can be unsafe from that point of view*.

[D003]

Participants noted the central tension that reading others’ experiences could be both challenging and helpful, necessitating a selective approach to what they access.

*Some of the content was difficult to read, especially given what I was going through and how I felt but it was quite nice to be able to see other people who could identify with posts and stuff saying, “It’s not just you, this is me too.” So, my experience is a bit mixed, but I picked my diet so that’s on me to a certain extent.*

[D090]

*...with your safety I think for me it’s keeping it simple and definitely putting your boundaries down and saying, this is acceptable, this is not acceptable*...

[M100]

Examples of forum features that could support this form of self-moderation included content warnings.

*...they always have a warning at the top which is good because if you are sensitive that kind of stuff, you can always not view it, you have a choice at that point*.

[D073]

Forums set up in such a way that the main text of a post was hidden also supported this decision-making process.

*You don’t have to actually delve into reading what they’ve put ... You might just get the header and think I won’t bother reading that so that’s why I don’t bother because I think I already know them, that’s going to upset me, so I’m not going to...*

[M126]

### CMO5: Interpersonal Safety

#### CMO Configuration

##### Context

Forum users, particularly those early in their forum experience, may be worried about viewing harmful online content and not being welcome in the community.

##### Mechanism

Seeing forum posts containing authentic accounts of shared lived experiences and posts that communicate an attitude of nonjudgmental acceptance (resource) facilitates a feeling of trust that community members are safe to share personal experiences with (response).

##### Outcome

Psychological safety, which in this context refers to feeling that the forum is a place where users feel able to bring their own views and experiences to the community.

### Illustrative Interview Data

One user recognized that others posting about their lived experiences contributed to a sense of safety that promoted the reciprocal exchange of personal difficulties.

*... Even though you don’t know who each other are it’s safe knowing that other people are going through the same things and they’re almost trusting you as much as you’re trusting them just to open up that little bit to try and get through whatever you’re getting through.*

[D196]

The following participant identified that those sharing authentic accounts of mental health challenges would be less likely to judge those going through similar experiences.

*Interviewer: what makes a forum feel safe to talk about mental health?*

*Respondent: I’d say that making sure the comments are like they feel authentic I think and that’s what Dunnock can ... you’re also talking to other people who are in similar situations and you can keep that aspect there I think it makes it feel a lot more authentic and safe to talk to because it’s more ... you don’t feel judged because they’re going through similar things.*

[D073]

A culture of acceptance further encouraged the following participant to feel able to discuss their mental health experiences online.

*Interviewer: Is there anything else that makes you feel that this is a place you can talk about mental health?*

*Respondent: It’s the general atmosphere ... it’s very accepting of anything you say like there’s nothing that would ... everyone would support you on anything you said, if you see what I mean. They wouldn’t, “That’s your fault,” or anything like that. I think it’s the atmosphere ... that this is very kind, supportive*...

[S006]

## Discussion

### Principal Findings

This study builds on findings from a previously published realist synthesis by highlighting and expanding on factors impacting users’ perceptions of safety in online mental health forums. Anonymity was found to promote disclosure by mitigating against online posts being linked to users’ offline identities, and by providing users with greater choice regarding what they share online. Perspectives on rule enforcement emphasized the importance of organizations balancing between proactive moderation to ensure that safety issues were addressed quickly and the need to intervene sensitively where conversations on restricted, yet potentially important personal experiences occurred. Participants reflected on the need to choose when and how to use online forums in light of their mental health, recognizing the possibility that accessing forum content could be helpful but also potentially detrimental at times. Factors contributing to a psychologically safe forum environment included the perceived authenticity of others in the community, an atmosphere of nonjudgment, and the value of sharing the space with others experiencing similar mental health difficulties.

### Comparison With Prior Work

Previous research using both qualitative interviews and analyses of forum posting behavior suggests anonymity is a key factor in forum safety [11,34]. The finding that the value of anonymity lies in avoiding the judgment of others aligns with the suggestion that online anonymity mitigates adverse consequences linked to personal disclosure, including reputational harm, social pressure, and exclusion [35]. While in-forum anonymity appears broadly valued by users, variations in forum design highlight important choices regarding its implementation. Some forums emphasize complete anonymity such that users are unknown to each other and the host organization, while other platforms may collect personal information in the interest of safeguarding. Moderation practices also vary. Some forums prioritize online privacy and remove identifying information, while others may allow the sharing of some personal details, which as one participant noted here, can facilitate social connection. In balancing the advantages and challenges associated with anonymity, forum designers may need to consider relevant legal, ethical, and practical issues. These include legal obligations regarding the protection of personal data, the organization’s responsibility for user well-being, community members’ awareness of the implications of sharing their details online, and whether the forum is closed or open to the public [15,36]. Whether differences in the implementation of anonymity impact willingness to register or post to online forums is a potentially valuable avenue for further research.

Participants’ reflections on content moderation emphasized the value of proactivity in managing problematic content, and interpersonal sensitivity when intervening to implement rules. Results here highlight the need for forums to balance between the benefits of open online discussion and the potential for censorship and disengagement. This topic has been the focus of extensive recent debate. Legal frameworks, such as the European Union Digital Services Act [24], have sought to place tighter rules on providers, whose responsibilities now include the need to implement and evidence content moderation and risk management procedures. These developments align with concerns that access to algorithmically generated and poorly managed social media content could rapidly expose users to high volumes of harmful content [37]. However, a recent academic debate on approaches to moderation revealed concerns that greater restriction on discussions of mental health difficulties represents a missed opportunity to facilitate supportive discourse, and creates undue barriers to support for those already experiencing limited access to care [38]. As part of this debate, researchers produced a call to action for more nuanced, evidence-based approaches to moderation. Suggestions included greater emphasis on community moderation, where site members’ knowledge of the community’s culture is leveraged to help make decisions about acceptable content, for example, via specific roles for experienced community members or the use of content labeling to allow users to more effectively flag harmful content [38].

A novel contribution of this study relates to users’ reflections on the need to self-moderate their access to online mental health forums. The recognition that forum content can be helpful and at times detrimental aligns with a large body of social media–related research highlighting both the advantages of social connection and the impact of distressing content, social comparison, and harmful advice [39]. Interviewees valued forum design features that supported their decisions to access or avoid potentially distressing content, such as content warnings, and forum providers may wish to consider additional strategies to support the user’s self-management of their services. Social media websites have in recent years integrated features including customizable update feeds and tools that filter content containing certain words or phrases, links to emotional support resources from trusted partners, and the use of content such as blogs to directly address the challenges of reading about distressing topics online [40]. To date, however, it is unclear to what extent these strategies are valued by users or effective in achieving their intended aims. Further research focusing on how services can support effective self-moderation of online communities is therefore warranted.

This paper emphasizes that moderators’ actions often underpin safe and effective online mental health forums. Existing research makes clear that intervening to support users and implement rules are highly skilled and demanding tasks. For example, the online context requires moderators to engage in a range of nuanced text-based practices to build rapport, understand users’ challenges accurately, and offer corresponding social support, including in situations where users are experiencing high levels of distress [41]. However, access to a large volume of distressing content, pressure to manage interpersonally sensitive and high-risk situations, and issues such as high workload, represent challenges to moderators’ workplace well-being [42,43]. Forum safety is therefore likely to be enhanced by sufficient support for moderators. This may include appropriate initial and ongoing training in the mental health difficulties likely to be discussed on the forum, support from other moderators, including during shifts, to share challenges and strategies for managing difficult situations, and supervision from senior colleagues [19].

Online forums rely on a regularly updated stream of user-generated content, yet only a minority of those viewing forums post to them [44]. It is therefore vital to understand factors that may promote engagement with online conversations. Results here suggest that positive perceptions of the interpersonal safety of online forums may promote engagement and that this relies on a community embodying nonjudgment and being populated by people with similar mental health experiences whom forum users may intuitively trust with their own narratives. As previously noted, the concept of psychological safety, typically defined as a culture in which people feel able to take interpersonal risks, has parallels with this aspect of forum user experience. Consistent with the findings reported here, trust has been identified as an antecedent to psychological safety in online communities [20]. While largely conducted within in-person and nonclinical groups, psychological safety research has demonstrated that its presence is associated with knowledge sharing and “speaking up” behaviors, and its absence with “silencing behaviors” rooted in fear of negative social consequences [45]. Given that the psychological safety construct has been fruitfully applied in occupational research to identify individual and group-level factors linked to a range of important outcomes, including learning, well-being, and workplace performance, it may be advantageous for future research to apply this conceptual lens in developing a more thorough understanding of forum safety.

### Limitations

This study has several limitations. First, the sampling strategy recruited current users of online mental health forums. This study therefore does not capture the perspectives of those who no longer use these services, some of whom may not do so because of fears over safety, and as such, this sample may provide more insight into safety-promoting measures rather than actions forums hosts should avoid. Second, participants were recruited from UK-based online mental health services focused on particular mental health challenges, potentially limiting the transferability of our findings to other forum contexts. A third related issue is that while all of the experiences described in the IPT framework were probed as part of the realist interviews, some, such as the negative impacts of anonymity and the normalization of harmful behavior, did not emerge as prominent issues in interviews with the participants in this study and were therefore not reflected in the refined program theories. This may be because forums used for recruitment to this study were actively moderated services, limiting exposure to these experiences.

### Conclusions

Understanding how to deliver safe online mental health support is a topic of growing international significance. The findings of this realist evaluation highlight how users’ perceptions of safety depend both on features of forum design—such as anonymity and content moderation—but also on the interpersonal interactions that emerge from their implementation. Delivering safe and effective online mental health forums therefore depends on understanding how, in practice, steps taken to reduce harm and promote engagement are experienced, highlighting the need for ongoing research in this domain.

### Lived Experience Commentary

A lived experience commentary by an expert is presented in Textbox 1.

Textbox 1. **Lived experience commentary by Sophia Tai.**

The research in this realist evaluation showed not only do mental health forums provide safety and an antidote to loneliness but also have the potential to cause harm and distress to its users if moderated poorly without the correct parameters. Moderation can be applied with the help of trained moderators or by self-moderation such as content filter settings to avoid triggering posts that may be harmful or distressing. However, while I agree that self-moderation is essential for user autonomy, it is not always enough. A mental health forum would imply that users who join may not always be in their best mental or emotional state, making them more vulnerable. From personal experience, any level of self-moderation can start showing gaps as our mental health starts to suffer. One example forum mentioned in this paper also supports teenagers under the age of 16. I would argue that effective self-moderation requires a level of maturity not necessarily present in this age range. With this in mind, I urge the importance of investment in highly trained moderators which this paper highlights as a key element for safeguarding mental health forum users.

This paper pointed out the accessibility of mental health forums, which helps overcome time barriers such as long NHS waitlists or financial barriers such as private therapy costs. Not being able to access immediate support can cause an individual’s condition to get worse quickly. Having access to a safe forum can provide effective support to a large demographic, or those with barriers to immediate support. I agree with the findings in this research that a forum with poor or no moderation is worse than not joining a forum at all. There needs to be a support system in place to ensure moderators can maintain good mental health and remain in a strong supporting role for those who are vulnerable. A well-moderated forum would not only support a larger demographic in the mental health sphere but also give intermediate support for those on waitlists or aftercare for those coming out of sessions and finding their own footing after mental health care.

## Supplementary material

10.2196/75320 Multimedia Appendix 1 Interview topic guide.

## Abbreviations

CMO: context-mechanism-outcome
iPOF: Improving Peer Online Forums
IPT: initial program theory

## References

[1] Torous J, Bucci S, Bell IH (2021). The growing field of digital psychiatry: current evidence and the future of apps, social media, chatbots, and virtual reality. World Psychiatry.

[2] Naslund JA, Bondre A, Torous J, Aschbrenner KA (2020). Social media and mental health: benefits, risks, and opportunities for research and practice. J Technol Behav Sci.

[3] O’Grady L, Bender J, Urowitz S, Wiljer D, Jadad AR (2010). Promoting and participating in online health forums: a guide to facilitation and evaluation for health professionals. J Commun Healthcare.

[4] Rayland A, Andrews J (2023). From social network to peer support network: opportunities to explore mechanisms of online peer support for mental health. JMIR Ment Health.

[5] Marshall P, Booth M, Coole M (2024). Understanding the impacts of online mental health peer support forums: realist synthesis. JMIR Ment Health.

[6] Rice S, Gleeson J, Davey C (2018). Moderated online social therapy for depression relapse prevention in young people: pilot study of a ‘next generation’ online intervention. Early Intervention Psych.

[7] Yeo G, Loo G, Oon M, Pang R, Ho D (2023). A digital peer support platform to translate online peer support for emerging adult mental well-being: randomized controlled trial. JMIR Ment Health.

[8] Newman A, Donohue R, Eva N (2017). Psychological safety: a systematic review of the literature. Hum Resour Manage Rev.

[9] Stana A, Miller AR (2019). “Being a mom = having all the feels”: social support in a postpartum depression online support group. Atl J Commun.

[10] Siling JL, Truss K, Philips L, Eastwood O, Bendall S (2021). Young people’s journeys of recovery from trauma: a qualitative study of narratives from Internet forums. Psychol Trauma.

[11] Smit D, Vrijsen JN, Groeneweg B, Vellinga-Dings A, Peelen J, Spijker J (2021). A newly developed online peer support community for depression (depression connect): qualitative study. J Med Internet Res.

[12] Easton K, Diggle J, Ruethi-Davis M (2017). Qualitative exploration of the potential for adverse events when using an online peer support network for mental health: cross-sectional survey. JMIR Ment Health.

[13] Mokkenstorm JK, Mérelle SYM, Smit JH (2020). Exploration of benefits and potential harmful effects of an online forum for visitors to the suicide prevention platform in the Netherlands. Crisis.

[14] Bailey E, Alvarez-Jimenez M, Robinson J (2020). An enhanced social networking intervention for young people with active suicidal ideation: safety, feasibility and acceptability outcomes. Int J Environ Res Public Health.

[15] Sharkey S, Jones R, Smithson J (2011). Ethical practice in internet research involving vulnerable people: lessons from a self-harm discussion forum study (SharpTalk). J Med Ethics.

[16] Suarez-Lledo V, Alvarez-Galvez J (2021). Prevalence of health misinformation on social media: systematic review. J Med Internet Res.

[17] Arora A, Nakov P, Hardalov M (2024). Detecting harmful content on online platforms: what platforms need vs. where research efforts go. ACM Comput Surv.

[18] Bailey E, Robinson J, Alvarez-Jimenez M (2021). Correction: moderated online social therapy for young people with active suicidal ideation: qualitative study. J Med Internet Res.

[19] Robinson H, Booth M, Fothergill L (2024). Understanding the needs of moderators in online mental health forums: a realist synthesis and recommendations for support (preprint). JMIR Ment Health.

[20] Zhang Y, Fang Y, Wei KK, Chen H (2010). Exploring the role of psychological safety in promoting the intention to continue sharing knowledge in virtual communities. Int J Inf Manage.

[21] Gibson K, Trnka S (2020). Young people’s priorities for support on social media: “it takes trust to talk about these issues”. Comput Human Behav.

[22] Paulus TM, Varga MA (2015). “Please know that you are not alone with your pain”: responses to newcomer posts in an online grief support forum. Death Stud.

[23] Trengove M, Kazim E, Almeida DRS, Hilliard A, Lomas E, Zannone S (2022). A digital duty of care: a critical review of the online safety bill. SSRN J.

[24] Turillazzi A, Taddeo M, Floridi L, Casolari F (2023). The digital services act: an analysis of its ethical, legal, and social implications. Law Innov Technol.

[25] Lobban F, Coole M, Donaldson E (2023). Improving Peer Online Forums (iPOF): protocol for a realist evaluation of peer online mental health forums to inform practice and policy. BMJ Open.

[26] Tilley N, Pawson R (2000). Realistic Evaluation: An Overview.

[27] Hunter R, Gorely T, Beattie M, Harris K (2022). Realist review. Int Rev Sport Exerc Psychol.

[28] IPOF forum summaries. Lancaster University.

[29] IPOF ethics framework. Lancaster University.

[30] Shryane N, Glossop Z, Jones S (2024). iPOF survey protocol.

[31] Manzano A (2016). The craft of interviewing in realist evaluation. Evaluation (Lond).

[32] MHPRU lived experience commentary. UCL.

[33] Jagosh J, Stott H, Halls S (2022). Benefits of realist evaluation for rapidly changing health service delivery. BMJ Open.

[34] De Choudhury M, De S (2014). Mental health discourse on reddit: self-disclosure, social support, and anonymity. ICWSM.

[35] Pan X, Hou Y, Wang Q (2023). Are we braver in cyberspace? Social media anonymity enhances moral courage. Comput Human Behav.

[36] Khare S, Kalamkar M (2023). Safeguarding Online Confidentiality and Security in the Age of Social Media.

[37] Russell I (2024). Debate: more, not less social media content moderation? How to better protect youth mental health online. Child Adolesc Ment Health.

[38] Zhang CC, Zaleski G, Kailley JN (2024). Debate: social media content moderation may do more harm than good for youth mental health. Child Adoles Ment Health.

[39] Khalaf AM, Alubied AA, Khalaf AM, Rifaey AA (2023). The impact of social media on the mental health of adolescents and young adults: a systematic review. Cureus.

[40] What are the social media platforms doing to support users’ mental health?. Social Day.

[41] Perry A, Christensen S, Lamont-Mills A, Du Plessis C (2024). Keeping users experiencing a suicidal crisis safe online: current text-based practices of professional online mental health forum moderators. Cyberpsychology (Brno).

[42] Deng D, Rogers T, Naslund JA (2023). The role of moderators in facilitating and encouraging peer-to-peer support in an online mental health community: a qualitative exploratory study. J Technol Behav Sci.

[43] Steiger M, Bharucha TJ, Venkatagiri S, Riedl MJ, Lease M The psychological well-being of content moderators: the emotional labor of commercial moderation and avenues for improving support.

[44] Wilkerson DA (2016). Lurking behavior in online psychosocial discussion forums: theoretical perspectives and implications for practice. J Technol Hum Serv.

[45] Edmondson AC, Bransby DP (2023). Psychological safety comes of age: observed themes in an established literature. Annu Rev Organ Psychol Organ Behav.

