# Supplementary material for: Understanding Safety in Online Mental Health Forums: Realist Evaluation
Source: JMIR Ment Health. 2025 Jun 27;12:e75320. doi: 10.2196/75320 (PMC12227176; doi:10.2196/75320)
Supplement: Multimedia Appendix 1 [file mental-v12-e75320-s001.docx]

**Improving Peer Online Forums**

**Workstream 2 Interview Topic Guide**

**Introductory questions**

- Which forum(s) do you use?
- What do you tend to use the forum for?
- What motivated you to start using it?
- How frequently do you use it?

**Topic specific questions – Psychological safety**

*Exploratory questions:*

- We’re interested in what makes a forum feel like a safe space. What makes a forum safe enough for you to discuss and read about issues like mental health?

*Theory-informed questions:*

- Do forum rules influence how safe the forum feels?
  - Are there any specific rules/examples that come to mind?
- Do moderators influence how safe the forum feels?
  - Can you think of any examples of things moderators do to promote safety?
- Some research shows that people might come across things on forums that they find upsetting. Have you ever experienced that?
  - How did you react?
  - Did that influence how much you used the forum
- One idea from previous research is that people tend to stay on forums when they feel welcomed into the online group. What is your view of that idea?
  - What influenced how welcoming the forum felt when you joined?
  - Have you ever used a forum that felt unwelcoming? What was your reaction?
- Some forums have ways to highlight that posts are about sensitive topics, for example there may be sub-forums dedicated to sensitive issues, tags on posts, or trigger warnings. Does the forum have those features?
  - Does this influence how safe the forum feels?
- Some previous research suggest that online forum conversations could encourage behaviours that could be harmful, such as self-harm or restrictive eating. Is that something you have come across online?
  - What was your reaction?
  - how is that managed by the forum/organisation?
- Some people report that they started to post in online forums because they saw other people getting friendly and helpful responses. What is your view of that?
- How does the ability to post anonymously influence the way you use the forum?
